# Surface electromyography onset-based processing for control of a robotic arm

**DOI:** 10.3389/frobt.2026.1968921

**Published:** 2026-10-26

**Authors:** John Niezgoda, Apostolos Kalatazis

**Affiliations:** 1 Department of Mechanical Engineering, Cleveland State University, Cleveland, OH, United States; 2 Department of Computer Science, Cleveland State University, Cleveland, OH, United States

**Keywords:** electromyography, motion, prosthesis, robot, signal processing

## Abstract

Upper-limb amputation can have a significant impact on a patient’s quality of life, including limited independence. While myoelectric prostheses present the opportunity for more natural and intuitive control when compared to body-powered devices, their effectiveness can be limited by the challenges that come with surface EMG data collection. This study proposes a simplified EMG post-processing approach for converting muscle activation into robotic arm movements. Surface EMG data was collected sequentially from ten locations on the upper limb of a single subject that was instructed to perform four predefined motion sequences designed to cover common upper limb motions. The signals were processed via a custom pipeline that includes filtering and onset detection using NeuroKit2. The muscle activations detected were then mapped to commands for a Baxter robotic arm, which was used as a proxy for a prosthetic device. The resulting robotic motions were compared to the human subject’s via the use of joint angle measurements and the time to complete each sequence. The results show that the proposed pipeline can reproduce the general structure and range of human motion, with the robot achieving joint movements similar to those of the participant. However, the robot suffered from a longer time required to complete each sequence of movements. This study shows that a relatively simple post-process EMG-based control scheme can capture multi-step human motion while remaining transparent. However, improvements in speed and real-time performance will be necessary for practical use in prosthetic devices.

## 1 Introduction

The loss of a limb’s function can be devastating for a patient. In the case of traumatic upper-limb amputation, this can result in pathological grief and feelings of functional impairment (Pomares et al., 2020). In the United States, estimates place the prevalence of patients with amputations at around 1.6–1.7 million with expectations for the number to more than double by 2050 (Pezzin et al., 2004; Ziegler-Graham et al., 2008). In a review of 19 studies, the cause of upper limb amputations was found to predominantly be trauma, but a not-insignificant amount were caused by medical conditions such as diabetes (Choi et al., 2025). One study found that many patients may experience difficulties performing everyday tasks, with the majority of upper-limb amputees expressing moderate or worse satisfaction with existing prostheses (Jang et al., 2011). As such, the critical functions of the upper-limb prostheses include the ability to perform grabbing actions reliably and without active supervision of the user (Cordella et al., 2016).

One solution for improving quality of life for an amputee is for the patient to be fitted with a prosthesis that can return some function of the amputated limb. One study of common daily tasks found that the majority of participants performed them bimanually (Kilbreath and Heard, 2005). Restoring partial or full functionality of a second limb to a patient via a prosthesis has been shown to perform better on tasks than those who do not use one, allowing for greater independence (Resnik et al., 2020). Studies have shown that patients fitted with prostheses tend to utilize them more often with below-elbow (BE) amputations compared to above-elbow (AE) amputations (Wright et al., 1995; Millstein et al., 1982).

Despite these benefits, studies suggest prosthesis use among patients with upper limb amputation is approximately 60%–65%, with the causes of rejection attributed to perceived lack of function, lack of sensory feedback, or limited usefulness of the prosthesis (Wright et al., 1995; Resnik et al., 2023; 2019). One solution for some of these causes lies with myoelectric prostheses. These devices are powered via batteries or the like, and use nerve impulses or electromyogram (EMG) data to control the triggering of movements, in contrast to body-powered prosthesis relying on mechanic linkages. Research suggests the best performing prosthesis control method varies by user, with differences in realism and speed, with myoelectric devices tending to be slower than body-powered ones (Resnik et al., 2022; Elbasiouny, 2024; Farina et al., 2014). As such, a focus on improving the function of myoelectric prostheses may reduce rejection based on a perceived a lack of function. Myoelectric prostheses provide unique challenges, especially when controlled via surface EMG electrodes. While surface EMG data can be relatively simple to record, cross talk among adjacent muscles, the training required to accurately control such a prosthesis, and the potential recalibration of the limb due to changes in the electrode placement can provide barriers to intuitive use of this data (Elbasiouny, 2024). As such, improving the usability of surface EMG-based control remains a central challenge in the development of advanced upper-limb prostheses.

In this study, surface EMG data of a series of sequences of motions was recorded on the right arm using a series of predefined motion sequences to simulate motions to what a prosthesis user might desire. These sequences were chosen to include activation of muscle groups associated with manipulation of the hand and lower arm. To emulate control of a prosthesis, the recorded EMG data was mapped to joint commands for a robotic arm of the Baxter robot, manufactured by Rethink Robotics (2013). The Baxter is equipped with two arms that provide multiple degrees of freedom that can perform human-like motions, with only the left arm used in this study. The arm is equipped with a pincer gripper to replicate the basic grasping functions of a prosthetic hand. Using a robotic platform for testing and evaluating the data processing provides advantages to human testing, including the ability to iterate without the logistical constraints associated with human trials. The use of a static set of recorded data rather than live time series data allows for greater repeatability and removes the need for continuous participant involvement.

The primary contribution of this work is the development and evaluation of a post-process system of intuitive control that allows for multi-step motion sequences without the requirement of machine learning- or pattern recognition-based methods. The resulting pipeline uses activation onset detection and is human-readable and editable, while still reproducing the core human movements. Evaluating this approach using a Baxter robot against the original human motions provides insight into the effectiveness of surface EMG-based prosthetic control and offers a foundation for future development of improved myoelectric prostheses.

## 2 Background

Electromyography (EMG) signals are widely used in the control of human-like robotic systems, particularly the operation of prosthetic devices. These signals provide a measurable representation of the natural commands the body uses for motor function: neural impulses sent from the brain to muscles that result in the contraction of muscle fibers to generate mechanical forces (Farina et al., 2014; Sweeney and Hammers, 2018). This process occurs continuously in the human body, allowing complex motions to be performed with little conscious effort. During this motion, the neural impulses generate electrical activity within the muscle tissue. EMG measures this activity, whether via surface electrodes placed on the skin or by more invasive methods such as needle electrodes that can be inserted directly within muscles. The recorded EMG signal is the sum of the electrical potentials produced by the individual motor units that are recruited when the muscle contracts (McManus et al., 2020). Due to these signals being closely related to the neural commands responsible for the activation of the muscle, they give valuable, recordable insight into a patient’s intended motion. The relative ease of capturing and interpreting EMG signals has therefore made them an attractive method for building human-machine interaction (Esposito et al., 2021). In the case of prosthetic systems, these signals can be used to infer the motion of a missing limb, build more natural human motion into a prosthesis, or make control of the limb more intuitive. Similarly, EMG-based systems are used in a wide variety of robotic applications, including exoskeletons, assistive devices, and rehabilitation technology (Hays et al., 2023; Li et al., 2021). These systems aim to utilize the natural signals produced by the user to create improved control mechanisms when compared to traditionally available mechanical or manual interfaces. Despite this potential, using EMG signals for controlling devices presents several technical challenges. EMG signals are low in amplitude, can be susceptible to noise and artifacts, and must be processed to extract useful information (Clancy et al., 2002). Therefore, for successful EMG control, two fundamental components are required: accurate acquisition of the EMG signals and the development of processing techniques that can convert these signals into usable inputs for the controlled device.

### 2.1 EMG data collection

The collection of EMG data can be conducted using several acquisition methods, broadly divided into surface and subcutaneous approaches. Surface EMG is commonly used due to it being non-invasive and the relative ease of application. Surface EMG sensors are typically placed directly on the patient’s skin above the muscle that data is desired to be collected from and will detect electrical potentials that are generated within the surrounding tissue. There are several different methods of surface EMG devices, that include bipolar electrodes, adhesive contacts, or grids of contacts (Merletti and Muceli, 2019).

In contrast to the surface EMG devices, subcutaneous EMG devices involve placing electrodes beneath the patient’s skin to measure muscle activity more directly. Some methods include intramuscular EMG and fine-wire electrodes, which are inserted directly into the muscle tissue. Once positioned, these electrodes are able to detect the EMG signals with a high degree of specificity (Chae et al., 2001). This makes subcutaneous EMG especially useful for studying the behavior of motor units and gaining more detailed insight into the muscles. However, as the electrodes must penetrate the skin and muscle tissue, subcutaneous methods are invasive and will typically require trained personnel to perform the insertion, reducing the viability of subcutaneous EMG for non-clinical settings.

As such, utilizing surface EMG techniques offers several advantages. They are non-invasive and therefore more suitable for repeated sessions of measurements, long durations of recordings, and studies involving larger numbers of participants. Surface electrodes can be applied quickly without the need for specialized medical training. This makes surface EMG devices much more practical for experiments, rehabilitation, and wearable technology. However, due to the sensing electrodes being physically further away from the muscles producing the potentials they are trying to record, the resulting signals can contain greater levels of noise (Journée et al., 2020). Additionally, the signals being recorded from the surface of the limb may result in the EMG device detecting and including signals from nearby muscles, known as crosstalk (De Luca and Merletti, 1988).

Regardless of the recording method utilized, the data is typically represented as a time series of the voltage changes detected over time. These signals are often sampled at high frequencies, commonly ranging from approximately 1000–4000 Hz to attempt to accurately capture fluctuations in muscle activity (BIOPAC Systems, Inc, 2026; Delsys Incorporated, 2023; Noraxon U.S.A. Inc, 2025). Due to the nature of how motor units are recruited in the muscle to produce a desired motion, raw EMG signals tend to be somewhat random and unpredictable waveforms. In addition to other factors such as noise and crosstalk, the raw signals must usually be processed before useful analysis can be conducted.

### 2.2 EMG data processing

EMG data processing pipelines consist of several stages: acquisition of data, preprocessing or filtering, and classification of data or mapping to the desired control outputs. The preprocessing and filtering stage focuses on preparing data for later analysis. Classification translates processed signals into commands that correspond to the subject’s desired movements. Having a well-designed pipeline that supports the intended application is critical, as the reliability and responsiveness of the prosthesis depends heavily on the interpretation of the recorded signal.

EMG signals are inherently random and susceptible to noise, thus it is critical to preprocess the signal prior to analysis with steps designed to remove the noise and improve the quality of the signal (Clancy et al., 2002). Common techniques include band-pass filtering to eliminate low-frequency artifacts and smoothing algorithms such as moving average or root mean square (RMS) methods to generate more stable signals for later analysis (Piyathilaka et al., 2026). Common tools have been developed that can help both simplify and standardize the way data is processed, including NeuroKit2, which aims to offer an open-source library for processing EMG and other neurophysiological data (Makowski et al., 2021). NeuroKit2 has been cited in similar works investigating electromyography (Farook et al., 2024; Ehkirch et al., 2025). This tool offers a convenience function for automatically processing EMG signals, including the output of cleaned and fully preprocessed signals and onsets and offsets of the muscle activations. Tools like NeuroKit can help improve both transparency in data processing and reproducibility of the results. Once processing of the data is complete, it must be analyzed to provide useful information for analysis or control of a robotic system.

A variety of methods have been proposed for the mapping of EMG data, ranging from relatively simple direct control schemes to more advanced pattern recognition approaches. Direct control ties specific EMG signals or activations to the actuation of a motor or device using simple thresholds or mappings. In contrast, pattern recognition does more complex analysis of the data to detect additional insights, such as the type or strength of the motion recorded.

Pattern recognition generally uses a machine learning model trained on large quantities of data that can map patterns across EMG signals to specific commands, potentially allowing more intuitive control of a prosthesis. Pomares et al. (2020). This method can provide challenges, including the large and patient-specific data set required due to the nature of EMG signals varying between individuals (Zhao et al., 2021). Even individual factors such as muscle fatigue, perspiration, and minor displacement of electrodes can complicate the training, resulting in a time-consuming process (Kyranou et al., 2018).

An alternative approach is the simpler direct control method, where the amplitude or onset of the EMG signal is used to directly change the speed or position of a joint on the prosthetic limb. For example, a muscle group may be assigned to control the opening of the hand when activation of that group is detected. This method relies on more straightforward signal processing, such as thresholding or onset detection, requiring a smaller quantity and less individual-specific data. This can give direct control methods an advantage over pattern recognition. One study comparing the use of pattern recognition vs. direct EMG control found that the users with direct control performed better on tasks than pattern recognition, especially related to dexterity (Resnik et al., 2018). However, this may be the result of subjective preferences, as a similar study found that subjects preferred the use of pattern recognition control, with performance increasing with more familiarization (Hargrove et al., 2017).

### 2.3 Similar work

Previous work has demonstrated a wide range of EMG-focused robot control strategies, including direct control and machine-learning methods. These approaches generally seek to improve classification accuracy, provide greater degrees of control, and behave in intuitive manners to the user. However, this often comes at the cost of increased computational complexity and the scale of data sets required.

Artemiadis and Kyriakopoulos (2010) proposed a framework that accomplishes many of the same goals as this study, namely, creating an efficient method of converting EMG to robot control. This is done through the use of a mathematical dimensional-reduction technique for real-time control of the robotic limb. However, this model does require a limited amount of subject-specific training and model calibration. Most importantly, it focuses on capturing the motion of the shoulder, upper arm, and forearm, comparing the human to robot hand or end effector position via coordinates in a three-dimensional space, without the inclusion of wrist or hand manipulation. While the study’s proposed model may be effective for overall arm motion, control of a hand or end effector of a robotic arm is important for the viability of a prosthetic limb, as discussed in the introduction. Our work investigates a method that does not require subject-specific training and uses a simpler, onset-based technique to allow control of the robot’s end effector.

A different study, Hassan et al. (2020), suggests the use of a myoelectric armband around the forearm to record and process data through a pattern recognition system to control a robotic arm with the advantages of a wireless, adhesive-less method of collecting EMG data. While this system does include the function of the hand and wrist, it is limited due to the armband only being able to sense the muscles within its physical footprint, such as those controlling the flexion or extension of the elbow. Additionally, the nature of the pattern recognition system required per-subject data collection and training of a model to accurately control the robot. Our study, via the use of an electrode system that allows for a wider range of position on the body, inherently has further data recording capabilities for the motion of the upper and lower arm and hand, again with the advantage of a general, onset-based approach.

In Vogel et al. (2011), the nature of machine learning or pattern recognition systems requiring large numbers of samples is further evident. The study proposed a functional system that enables upper and lower arm movement with hand control via the use of multiple sets of electrodes. However, the training data required to support this functionality consisted of thousands of samples, which were supplied by subsampling the training sets. While this method was successful, the quantity of data needed for pattern recognition methods were a deciding factor in our work focusing on simpler, more approachable methods.

Overall, the selection of the correct EMG signal processing strategy involves tradeoffs between the complexity of the system and motion desired, training data requirements, robustness, and performance. While pattern recognition offers more sophisticated control, direct control remains attractive due to its relative simplicity and reliability. selection must also be made in the wider context of the market for control schemes for prostheses, with other methods than EMG control being available.

## 3 Methods

Data collection for this study was conducted with a male, 22-year-old subject using the Biopac Systems, Inc. BioNomadix system. All experimental procedures were reviewed and approved by the Cleveland State University Institutional Review Board (IRB). Prior to participating in the study, the subject was informed of the testing procedures and provided written informed consent. The BioNomadix system utilizes three surface electrodes to measure a single EMG signal at a time. Due to this limitation, EMG signals were recorded sequentially rather than simultaneously, later being composited into a single file for unified processing. A total of 10 muscles of interest had EMG signals recorded over four unique motion sequences. The placement of the electrodes was on the surface of the skin, directly over the main body of the muscle of interest to target the maximum signal quality and reduce cross-talk as much as feasibly possible. Prior to the data recording session, the participant shaved their arm to remove the hair for better adherence of the electrodes, followed by a vigorous cleaning to remove skin oils and non-conductive dead skin cells. The electrodes were positioned according to the guidelines laid out by the ABC of EMG book, which specify a use of SENIAM procedures for applicable muscles, followed by placing the electrodes on the main muscle body along the direction of the muscle fibers (Konrad, 2006). Following placement, the electrodes were connected to the BioNomadix transmitter to capture muscle activation signals during each motion sequence. A list of the muscles of interest and the motion inferred upon a detected activation via the EMG signal can be found in Table 1. The data was recorded at a frequency of 2 kHz, with all settings in BIOPAC’s AcqKnowledge data acquisition program kept to the default. The electrodes were kept in place on a given muscle site for all trials of each motion sequence, then were removed and discarded. New electrodes were applied to the next muscle site, and again the data was recorded for each trial and motion sequence.

**TABLE 1:** Muscles of interest, positions, and associated movements.

**Position** #	**Muscle**/Muscle group of interest	Movement
1	Flexor carpi radialis	Controls wrist flexion
2	Extensor carpi radialis longus	Controls wrist extension
3	Pronator Teres	Pronates forearm (turn palm to face ground)
4	Supinator	Supinates forearm (turn palm to face up)
5	Extensor digitorum	Controls finger extension
6	Flexor digitorum superficialis	Controls finger flexion
7	Biceps brachii	Controls elbow flexion
8	Triceps brachii	Controls elbow extension
9	Brachialis	Controls elbow flexion
10	Brachioradialis	Controls elbow flexion

The muscles used were chosen were determined on two conditions: the ability of the muscle to be recorded by surface EMG electrodes and the motions produced by each muscle. For the direct control of the robot to be feasible, each movement determined must also have an opposite movement that can be recorded, otherwise only one direction of motion would be possible. For example, if there was a muscle whose signal triggers the robot’s E1 joint to rotate in the positive direction, there must also be a muscle that triggers the E1 joint to rotate in the negative direction. On examining the motion of the arm from the elbow down, four major pairs of motions were identified to be necessary for common motions: wrist flexion and extension, forearm pronation and supination, finger flexion and extension, and lastly elbow flexion and extension. The muscles that control these motions were therefore chosen to provide protagonistic and antagonstic constraints on each movement. Two additional muscles: 9, the brachialis; and 10, the brachioradialis; were also included in the data recording due to the ease with which they could be recorded. However, as they are redundant with the previous eight muscles chosen, they are not utilized in this study. The EMG signals from these two muscles are included in the published data set for completeness.

A total of four sequences of motions were recorded, covering finger, wrist, and biceps brachii flexion and extension, as well as wrist pronation and supination. These sequences were chosen to create a variety of combinations of the base, single-muscle motions that would be representative of potential use cases for a robotic arm. These motion sequences are described in Table 2. The weight used for Sequences one and two is approximately 1–2 pounds, such as a bottle of water, to give some slight resistance to the motion and an object for the subject to grasp. A metronome and timer were used to provide greater uniformity across the separate recordings required for each movement sequence. The metronome was set to 120 BPM, with a clear rhythm for the subject to follow as each motion was completed as a sequence of steps timed within that rhythm. For example, when the motion would call for the biceps brachii to be flexed, the subject would start the motion on the beat and flex their bicep as hard as they could in the time period between beats, finishing the motion on the next beat. This yields repeatable trials in terms of time and preserved the timing at which muscle activations occurred, which is necessary for this study due to each signal being recorded sequentially.

**TABLE 2:** Descriptions for the steps of each sequence.

Sequence	1	2	3	4
Initial position	Participant starts in base position with a weight where the hand can grasp it		Participant starts in base position but with the wrist pronated such that the palm is flat against the table. There is no weight	
Step 1	Fingers are flexed to grasp the weight	Fingers are flexed to grasp the weight	Fingers are flexed to close the hand into a fist	The wrist is supinated such that the back of the palm is now against the table
Step 2	Bicep is flexed to bring the forearm vertical, against upper arm	The wrist is pronated to have the palm and weight parallel to the table surface	Bicep is flexed to bring the forearm vertical, against upper arm	Bicep is flexed to bring the forearm vertical, against upper arm
Step 3	Wrist is flexed (and stays flexed)	Fingers are extended to release the weight	Fingers are extended to open the hand fully	Wrist is flexed so the fingertips touch the shoulder
Step 4	Arm is extended until the forearm is again parallel to the floor	A brief pause is taken where muscles are relaxed, but position stays the same	Arm is extended until the forearm is again parallel to the floor	Wrist is extended so the palm is vertical
Step 5	Fingers are extended to release the weight	Fingers are flexed to grasp the weight		The wrist is pronated such that the palm faces outwards
Step 6	A brief pause is taken where muscles are relaxed, but position stays the same	The wrist is supinated such that the palm and weight are now perpendicular to the table surface		Bicep is extended until the forearm is again parallel to the floor with the palm against the table
Step 7	Fingers are flexed to grasp the weight	Fingers are extended to release the weight		
Step 8	Bicep is flexed to bring the forearm vertical, against upper arm			
Step 9	Wrist is extended back to its original position			
Step 10	Bicep is extended until the forearm is again parallel to the floor			
Step 11	Fingers are extended to release the weight			

The raw data that was collected for this study has been uploaded to GitHub to a public repository. This data is free to access, and includes all the raw data files and the python programs written to process the EMG data. Each file is named in the form pAmBtC.csv, where A is the number of the muscle being recorded (see Table 1 for those pairings). B is the motion sequence being recorded, and C is the trial number for that particular recording. Thus, the file containing the data for the biceps brachii EMG signal during the fourth sequence would be found in the p7m4t1. csv file. Note that position 10, for the brachioradialis, uses X as the muscle number so the files are properly sorted.

The data is processed via the analysis_v5. py program, which can be found in its entirety in the same public GitHub repository as the raw data. It is written for Python 3.12. It requires a combined csv file of the raw EMG signal data for a given movement sequence from muscles numbers 1-8, as listed in Table 1. Additionally, the NeuroKit2 and Pandas libraries must be installed for the program to function properly. The goal of this program is to process the raw EMG signals such that a chronological triggering of motions for the Baxter robot’s arm can be output as a plain text list of values. The mapping between human muscle activations and the control of the Baxter robot was implemented by associating pairs of EMG-monitored muscles with specific joints on the robot. Each of the seven joints of the Baxter robot’s arms are labeled corresponding to their motion, as shown in Figure 1, with the addition of a gripper end effector. However, only three joints and the gripper were selected as control points for the myoelectric control system to allow for accurate control of both directions of rotation/function for each joint. The mapping of human motions to those performed by the robot can be found in Table 3. Each motion maps to setting a specific joint of the robot’s arm to a predetermined value. These angles are listed in Table 4. These angles were chosen by comparing the joint angles of a human in a given position and attempting to choose an angle that most closely approximates such a position on the robot. While the Baxter robot is an industrial-focused machine and not a prosthetic device, its construction allows for emulation of prosthetics due to its human-like jointing of its arms. When the joint E0 (shown in Figure 1) is rotated 180°, the lower joints on the limb provide analogues to the human body, including the elbow, wrist, and hand. Each motion and set of robot joint angles was tested prior to being combined in extended sequences Note that in this study, the EMG data was recorded on the participant’s right arm, but the robot motions were performed using the left.

**FIGURE 1:**
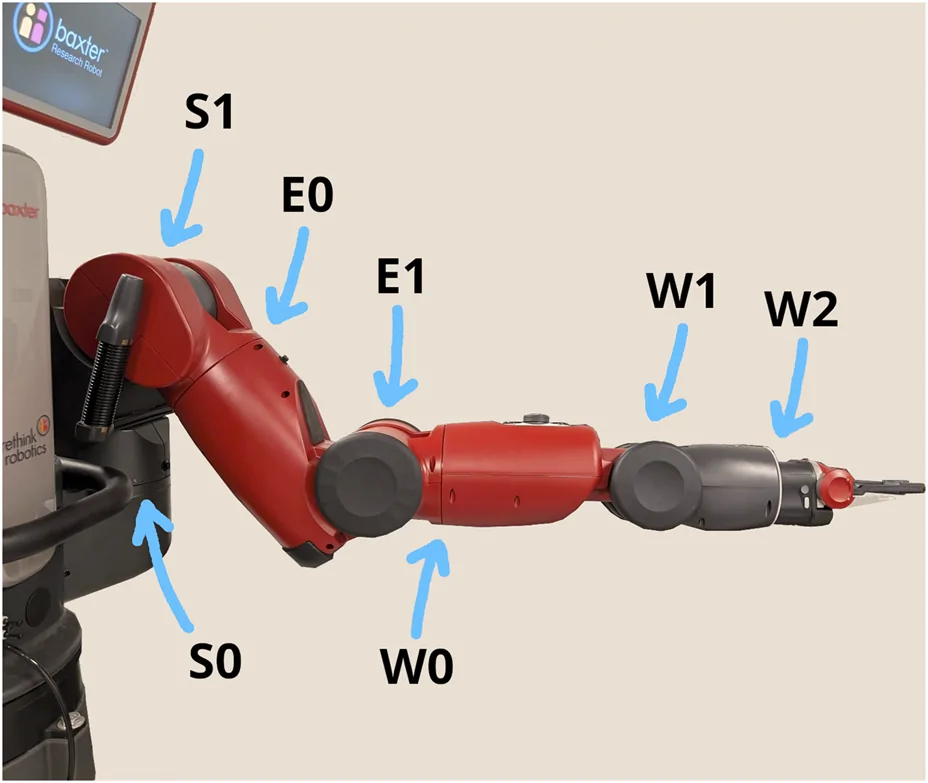
Joint labels for the Baxter robot’s left arm.

**TABLE 3:** Mapping of human to robot motions and motion codes.

**Motion** code	**Human** motion	**Robot** motion
1	Flex elbow	Rotate E1 in the positive direction
1.1	Extend elbow	Rotate E1 in the negative direction
2	Pronate wrist	Rotate w0 in the positive direction
2.1	Supinate wrist (hand vertical)	Rotate w0 in the negative direction, to base position
2.2	Supinate wrist fully (palm flat)	Rotate w0 in the negative direction past base position
3	Flex wrist	Rotate W1 in the positive direction
3.1	Extend wrist	Rotate W1 in the negative direction
4	Close hand (flex fingers)	Close gripper
4.1	Open hand (extend fingers)	Open gripper

**TABLE 4:** Robot joint angles.

Joint	**Initial** position	**Flexion**/ Pronation	**Extension**/ Supination
Left_s0	−0.78	-	-
Left_s1	0.78	-	-
Left_e0	3.14	-	-
Left_e1	0.8	2.5	0.8
Left_w0	1.57	3.0	1.57 or 0
Left_w1	0	1.57	0
Left_w2	0	-	-
Gripper	Open	Closed	Open

For each data signal, NeuroKit2’s emg_process function is used with a sampling rate of 2 kHz, and the amplitude and onsets of the processed signal are extracted. NeuroKit2’s emg_process function works as follows. It first cleans the signal using a fourth-order Butterworth high-pass filter with a 100 Hz cutoff frequency, applied using zero-phase forward-backward filtering, followed by constant detrending. No notch filter is applied. The cleaned EMG signal is then processed using the Teager-Kaiser Energy Operator (TKEO), with the resulting signal being filtered using a second-order Butterworth filter with nominal cutoff frequencies of 10 and 400 Hz, full-wave rectified, and the low-pass filtered using a second-order Butterworth filter with an 8 Hz cutoff to obtain the linear EMG envelope. This envelope is the amplitude of the EMG signal. Activation onsets were then extracted using the NeuroKit2 activation-detection procedure, with the minimum duration for an activation period being 50 m (Makowski et al., 2021). The version of NeuroKit2 used is 0.2.13.

With the processed signal and onsets, a minimum interval is defined for onsets close to one another, and onsets are filtered to remove those falling within the minimum interval. As the robot is controlled with a sequence of moves, this prevents the triggering of the same move twice in rapid succession from the program detecting multiple onsets from the same motion. The onsets for each signal are then sorted chronologically and mapped to the muscles.

The analysis_v5. py program outputs a string list of sequential movements for the robot to perform, which are then used as the input for the motion. py program that controls the Baxter robot. This program first sets the robot’s left arm to the neutral position. Note that for Sequences three and 4, an additional move is performed at the start of each move sequence to rotate the W0 joint such that the gripper is positioned like a palm-down hand. The program iterates through each motion in the move list sequentially until the sequence of moves is complete. Importantly, all EMG data is processed prior to the step of running the robot control program. The robot runs only with the output sequence of movements and does not process the EMG data during runtime. The output sequences were found to be the same across 10 different trials of each motion sequence, so only four unique sequences were generated to control the robot (one for each motion sequence). This suggests the processing method is consistent per the data recorded.

The joint positions of the robot were recorded to a ROS. bag file with time stamps. This data has an approximate frequency of 100–110 samples per second. The gripper’s state is recorded less frequently, at approximately 20 samples per second. The human joint angles were processed manually from recorded videos of the motion sequences. The videos were recorded at 60 frames per second at 4k resolution. The timestamps were determined as the frame at the start of the motion to the frame at the end of the motion. For each timestamped frame, the angle of the joints were measured via biological locations on the limbs, as shown in Figure 2. These timestamps and angles were determined for each sequence of motions. Two cameras were utilized: one from the side and another facing the subject’s front. Due to the order of moves in the output sequences being the same between trials for the recorded data, each motion sequence was run once to verify the sequence of moves and generate the required data for comparison. Multiple trials for the robot would not have varied the order of the motions.

**FIGURE 2:**
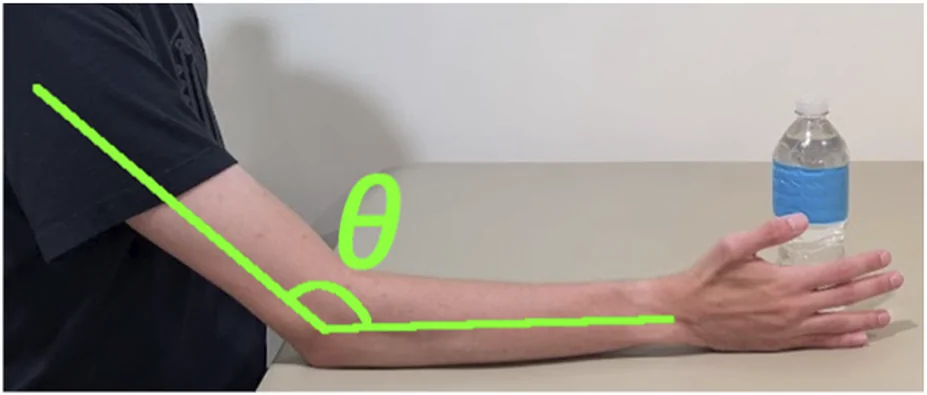
Method used for recording human joint angles; in this case, the biceps brachii.

## 4 Results

The joint angles in degrees and the gripper position for sequences one and four can be found in Figures 3, 6, next to the human measurements of the same sequence of motions. A key discrepancy between datasets arises from how gripper state is recorded. The robot’s gripper data, extracted from ROS bag files, only registers non-zero values when a closing command is issued. As a result, the data appears as discrete spikes rather than a continuous representation of gripper state over time. In contrast, the human hand state is represented continuously as a binary signal (0 = open, 1 = closed). This difference is especially apparent with the opening of the gripper, as that data was not logged in the ROS bag files, and does not appear on the graphs as a result. In Figure 6, for example, video recordings indicate that the gripper opens at approximately 53–54 s; however, this event is not reflected in the ROS data.

**FIGURE 3:**
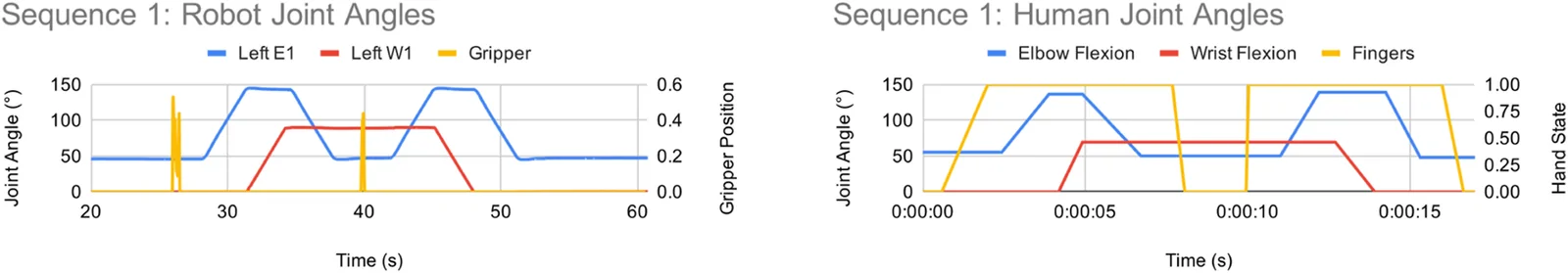
For sequence 1, the (Left) Robot joint angles; time scale is 40 s (20–60s). (Right) Human joint angles; time scale is about 16 s.

In Sequence 1, shown in Figure 3, the robot joint E1 corresponds to the elbow, W1 to the wrist, and the gripper to the opening and closing of the hand. The intended motion for this sequence is to pick up the object, bring the forearm vertical, flex the wrist, bring the forearm back to be parallel with the table, and release the object. Then, this process is completed in reverse order, as described in Table 2. With the human subject’s joint angles over time as a point of comparison, the robot was able to replicate the order of motions correctly. As the joint angles are predetermined values, as shown in Table 4, the joint angles produced are similar. Despite the Baxter robot recording only the movement of the gripper, these events, too, are represented in the correct order. The robot’s motions exhibit near-linear angular velocity for the majority of the motion, with some slight drift in the angle of the joint after the motion is complete. For example, at approximately 32 and 45 s, the movement of E1 is completed and the next movement of a different joint has begun, but the angle of E1 slowly decreases over the next 4 s until it again is affected by a movement.

In Sequence 2, shown in Figure 4, the robot joint W0 corresponds to the pronation and supination of the wrist and the gripper to the opening and closing of the hand. The intended motion for this sequence is to pick up the object, pronate the wrist, and release the object, then to complete the same moves in reverse order, as described in Table 2. Again comparing to the human subject’s joint angles over time, the order of motions was maintained. However, this sequence reveals some delay in the robot’s actions. No delay was programmed for waiting between motions on the robot save for delays of 2 s post-gripper actuation (to ensure full actuation of the gripper), but there is time delay between the W0 joint completing its rotation at about 23 s and the gripper actuating at about 26 s. While the final opening of the gripper at about 34 s is not shown due to the aforementioned data recording limitations, it seems that this same delay appears for the second rotation of W0, stretching from 30 s to 33 with an asymptotic approach to the final value.

**FIGURE 4:**
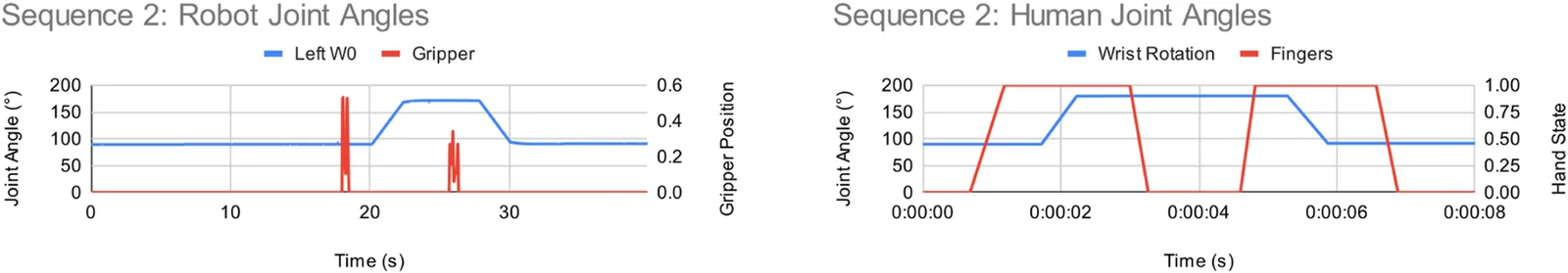
For sequence 2, the (Left) Robot joint angles; time scale is 40 s. (Right) Human joint angles; time scale is 8 s.

In Sequence 3, shown in Figure 5, the robot joint E1 corresponds to the elbow and the gripper to the opening and closing of the hand. The intended motion for this sequence is to pick up the object, flex the biceps brachii to bring the forearm vertical, release the object, and return to the start position, as described in Table 2. The joint actuation order is reproduced accurately when compared to the human order of motions, save for the omission of the data from the gripper at approximately 28 s. As this motion sequence is shorter, and the time scale on the figure is as well, there is a more clear representation of the motion characteristics of the robot’s non-gripper joints. The velocity of the joint when moving is mostly linear, but at the start and end of a motion, it exhibits some smoothing. The slight change in the position of the joint over the next few seconds after completing a move is clearly exhibited at about 27–29 and 32–34 s.

**FIGURE 5:**
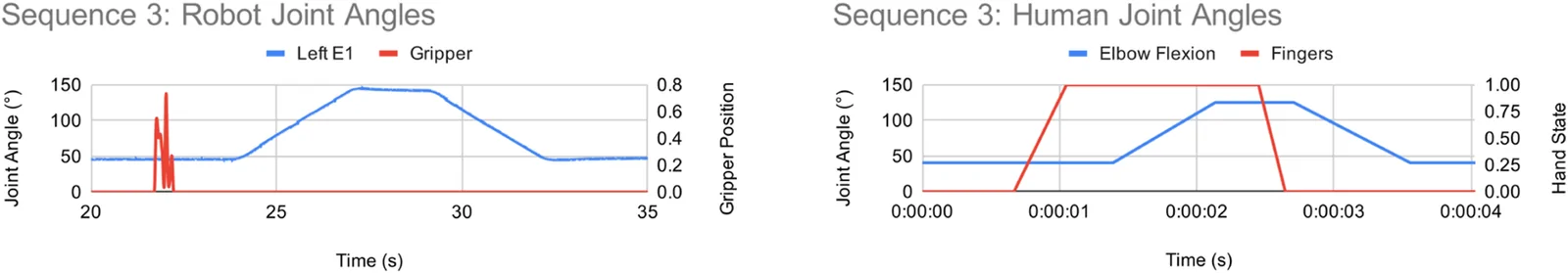
For sequence 3, the (Left) Robot joint angles; time scale is 15 s (20–35s). (Right) Human joint angles; time scale is 4 s.

In Sequence 4, shown in Figure 6, the robot joint E1 corresponds to the elbow, W0 to wrist pronation and supination, and W1 to wrist flexion and extension. The gripper is not used for this sequence. The intended motion for this sequence is to start with the hand and forearm flat on the table, turn the palm face-up, bring the forearm vertical, flex the wrist to touch the subject’s shoulder, extend the wrist back to its previous position, face the palm outwards, and then return to the original position, as described in Table 2. As with the previous sequences, the order of motions is maintained. However, it is important to note the gap of no movement per numerical data from about 39 to 52 s. In this time, the robot was attempting to move (the motors were engaged), but it was seemingly unable to complete the touch to the shoulder for several seconds. This lead to a long delay, after which the motion sequence resumed as normal. No delay was programmed for this step, rather, the robot’s internal code was not able to accept the position of the joint W1 as having completed the motion for some time.

**FIGURE 6:**
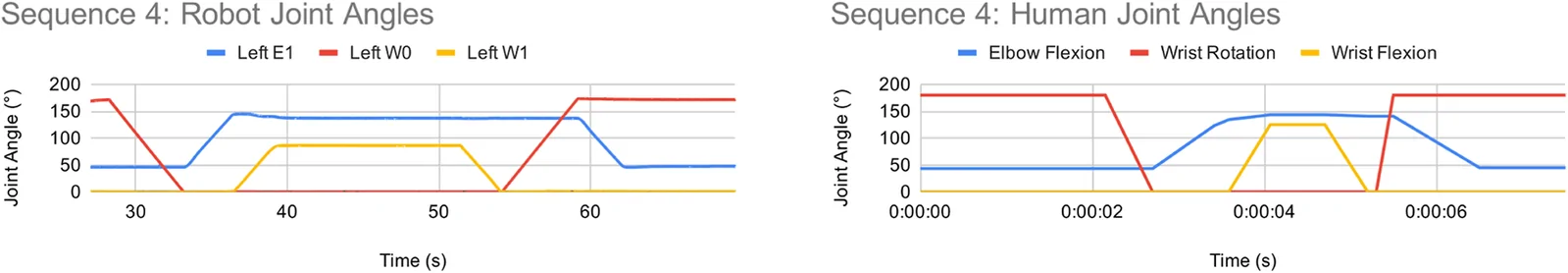
For sequence 4, the (Left) Robot joint angles; time scale is 55 s (25–80s). (Right) Human joint angles; time scale is 8 s.

Across all sequences, the robot required more time to complete each sequence, averaging 319% longer, than the human subject. The per-sequence timing comparisons are shown in Figure 7, with numerical values shown in Table 5. This discrepancy may be attributed in part to the robot’s fixed default speed, which was not adjustable in this study. However, the joint angle profiles may also have contributed, as shown in the robot’s motion in sequence two at about 23 s, see Figure 4. The Left W0 joint appears to move at a constant velocity for the majority of the motion, then asymptotically approaches the final value. The videos recorded of the Baxter robot motion show the asymptotic approach to the final joint angle as periods of near-zero velocity movement. A similar instance of this behavior can be seen in sequence four from 39 to 52 s, as shown in Figure 6. The robot seems to have reached about its final joint angle, but is not executing any moves for several seconds. This suggests that the robot may have built-in prioritization of motion smoothness and accuracy over speed. In contrast, the recorded human motion is characterized by much more abrupt motion, with faster transitions. In addition, it is important to note that the robot’s movement time is not affected by any processing of EMG data. EMG data processing happens in a separate, previous step to generate the move sequence. The move sequence is then iterated to directly control the robot. The time the robot spends completing a sequence is attributed to the real time taken to change a given joint angle, reach the desired value, and start the next move.

**FIGURE 7:**
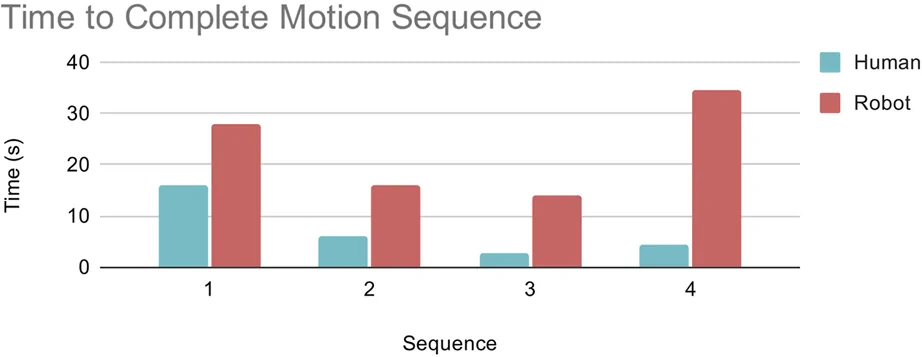
Comparison of sequence completion time between robot and human.

**TABLE 5:** Human vs. Robot Time to Complete Motion Sequences.

Sequence	**Human** (s)	**Robot** (s)
1	16.1	27.9
2	6.0	15.9
3	2.6	14.1
4	4.3	34.7

## 5 Discussion

This study’s results demonstrate a simplified EMG processing pipeline paired with a robotic arm is capable of reproducing human motion sequence order without requiring subject-specific machine learning models. This suggests that complex pattern recognition models may not be strictly necessary for creating functional prosthetic motion based on EMG data. A simplified approach has advantages in applications where interpretability and ease of implementation are prioritized and highlights the potential of direct control methods that do not require extensive user-specific training data. However, while the structural similarity between the human and robotic motions was maintained, a substantial discrepancy in execution time was observed. The results of this study are drawn from a single subject’s data, and so the results must be interpreted with the scope of this work being a feasibility study in mind.

### 5.1 Execution time

The robot’s execution of the move sequences was, on average, over three times longer than the human subject. While the Baxter robot seems to have motion characteristics that result in slower but precise movements, this is a large drawback in the use of this platform. As studies have shown, one of the unmet needs for prosthesis users is a lack of functionality (Resnik et al., 2023; 2022). A much slower time to complete tasks, therefore, represents a critical limitation in the practicality of the system. This may also help explain why simpler body-powered devices may be able to outperform myoelectric prostheses in certain tasks Farina et al. (2014). Robotic systems rely on control systems with tuned values, while a body-powered control scheme allows for perhaps less precision, but none of the overhead that a robotic control system requires. Without being able to modify the Baxter robot’s speed or control system, this gap may not be possible to close when using the Baxter as the robotics platform. Future research would require a platform that is more open to tuning the response of the control system. Reducing the precision of the robotic arm by allowing for faster movements with greater overshoot or a more specific tuning of the control system for this particular application could both reduce the execution time required to complete more advanced motion sequences while still maintaining consistent results.

### 5.2 Simplified pipeline

One of the core contributions of this study is the emphasis on the simplicity of the EMG processing pipeline. Many pattern recognition-based approaches rely on complex or opaque machine learning models to enable high-quality motion (Batzianoulis et al., 2018). While certainly beneficial for enabling complex motion to be reproduced, these pattern recognition systems suffer from the greater specificity of the model to each individual and the quantity of data required to train the classification algorithms (Zhao et al., 2021). Having a simplified pipeline built on standardized motions, as proposed in this study, removes this complexity and allows for less specific data requirements. While the use of a single participant limits the ability to generalize the findings of this study, as EMG signal characteristics vary between individuals and from electrode placement, the implementation of analysis based on muscle activation onsets is likely a more widely applicable method. Instead of relying on how a specific user’s EMG signals behave, merely the activation of the signal itself is the trigger for motions. As shown via the results of this study, building a complex, multi-step motion via the use of single-motion building blocks is feasible.

However, challenges with this method must be further investigated and solved before recommending the use of this simplified control method. Chiefly among them is the nature of the processing pipeline explored in this study being post-processing focused rather than a real-time implementation. The EMG processing methods used analyze the whole signal for activations rather than attempting to determine when an activation begins with real-time data. Additionally, a real-time system would necessitate simultaneous, multi-channel recording of EMG signals as opposed to the sequential collection of signals and later compositing of said signals. A real-time system would also introduce new constraints and challenges such as latency, which could affect the execution time or response of the robotic system.

### 5.3 Data limitations

When interpreting the results of this study, several limitations should be considered for further investigation. The study was conducted with a single participant, limiting generalizability across users. A wider sample size of subjects would help characterize the effectiveness of the pipeline presented in this study across different individuals. This would also help quantify the potential differences in the performance of this pipeline due to variances in subject muscle mass, body fat, and other biological differences. Different physiologies would also allow investigation into the effects of minor differences in electrode placement between sessions or participants.

Additionally, a major limitation of this study is the human joint angles. As they were processed manually via keyframes taken from video capture, there is potential measurement error and observer bias in the extraction of the joint angles. Future work involving human data capture would benefit from the inclusion of a more sophisticated motion capture system to enable more accurate and frequent joint angle data. Motion capture could also be used to fill the gap in the robot data for the gripper, as the log of joint states reported by ROS is unable to provide data on the position of the gripper, only recording when it is closing. This would allow for greater detail for both the human and robot motion data for directly comparing performance.

## 6 Conclusion

This study aimed to evaluate the feasibility of a surface EMG-based pipeline for translating human muscle activation into control of robotic arm motion. By mapping recorded EMG activations to joint commands for the Baxter robot, this study sought to determine how effectively the human movement sequences could be replicated using a simplified, human-readable control system. The results show that the proposed pipeline is capable of reproducing the overall structure and range of human motion with hardcoded joint angles, but the motion order being determined by the processed EMG data. These findings suggest that even a relatively simple control scheme approach mapping EMG signals to motions can preserve the essential characteristics of complex motion sequences. However, the longer time to execute these sequences, which took an average of over three times longer than the human subject, show a critical limitation of using a robot to simulate human motion.

In the context of upper-limb prosthetic devices, these findings reinforce the tradeoffs between control simplicity and function performance. While the proposed system offers transparency and clarity of processing, the reduced speed of motions may limit the practical application in real-world tasks. As perceived lack of function and limited usefulness of prostheses are major factors in rejection of the devices, improving the responsiveness of these systems remains an important requirement to address for user adoption.

Future work is necessary to transition this post-processing pipeline to one that can perform real-time control of a robotic device using simultaneous EMG signal acquisition. Investigating methods of changing the robotic control parameters, such as tuning the feedback system for moving to certain joint positions, may help improve the speed and performance of the robotic motion. Additionally, this study seeks to provide a methodology and proof-of-concept for improving the traits of prosthesis, and research directly involving prosthetic control with user data on these traits would be necessary to investigate usability, intuitiveness, or acceptance. Ultimately, this work highlights both the promise and challenges of myoelectric prosthetic control, suggesting that achieving intuitive, responsive, and reliable movement requires continued refinement of both data processing pipelines and robotic control strategies.

## Data Availability

The datasets presented in this study can be found in online repositories. The names of the repository/repositories and accession number(s) can be found below: https://github.com/j-niez/EMG-to-ROS.
